# p53 Protein Isoforms: Key Regulators in the Front Line of Pathogen Infections?

**DOI:** 10.1371/journal.ppat.1003246

**Published:** 2013-04-04

**Authors:** Olivier Terrier, Jean-Christophe Bourdon, Manuel Rosa-Calatrava

**Affiliations:** 1 Laboratoire de Virologie et Pathologie Humaine EA4610 VirPath, Equipe VirCell, Université Claude Bernard Lyon 1, Université de Lyon, Lyon, France; 2 Division of Medical Sciences, Centre for Oncology and Molecular Medicine, University of Dundee, Ninewells Hospital, Dundee, United Kingdom; The Fox Chase Cancer Center, United States of America

In response to extra- or intracellular stresses, the cellular gatekeeper p53 is able to integrate multiple diverse signals to determine the outcome of cell fate, by regulating the expression of numerous responsive genes [1], [2]. The tumor suppressor p53 has been extensively investigated in the field of cancer research [3], however there are reports it has a broader role in several other biological processes, such as metabolism, reproduction, or the immune response [1]–[4].

Bacterial and viral infections represent a major type of cellular stress, triggering different biological countermeasures, notably mediated by the p53 pathway. In the process of evolutionary adaptation to the host environment, pathogens have developed diverse strategies to hijack and exploit such host machinery. This is particularly well-illustrated for viruses, with many examples of virally induced deregulation of the p53 pathway (e.g., E1B of Adenoviruses; EBNA3C of Epstein-Barr virus, NS1 of influenza viruses) [5]–[7]. Similar interplays have also been reported for bacteria models, such as *Salmonella* or *Helicobacter* infections [8], [9]. The mechanisms and biological significance underlying regulation of p53 in the context of infection are not fully understood and often appear contradictory.

**Figure 1:**
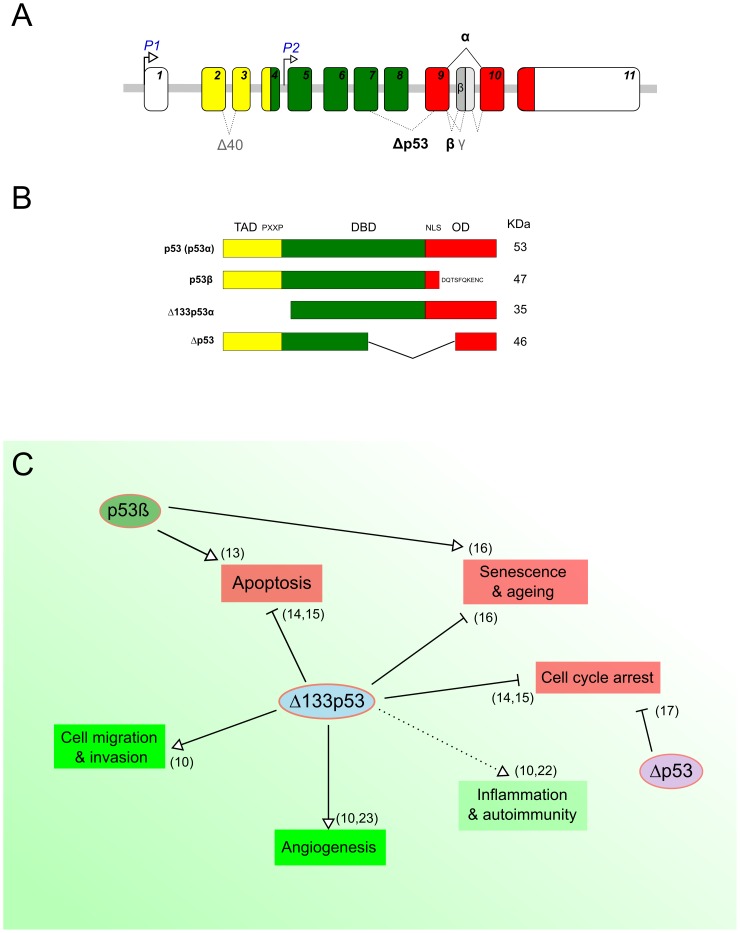
p53 protein isoforms and their biological functions. (A) Schematic representation of the human *TP53* gene. The human *TP53* gene contains 11 exons encoding several p53 products. The usage of the distal promoter (P1) leads to the production of p53 and Δ40p53 isoforms, while the internal promoter regulates the expression of Δ133p53 and Δ160p53 isoforms. (B) Schematic representation of some human p53 isoforms. The canonical p53 protein (p53α) contains a transactivation domain (TAD), a proline-rich domain (PXXP), a DNA binding domain (DBD), and a C-terminal domain—with a nuclear localization signal (NLS) and an oligomerization domain (OD). The C-terminal p53 isoform p53β is produced by an alternative splicing in intron 9, leading to the replacement of the OD by 10 new residues. The N-terminal p53 isoform Δ133p53 is encoded by a transcript initiated in intron 4 and lacks the TAD, PXXP, and part of the DBD. The Δp53 protein isoform is generated by a noncanonical alternative splicing between exons 7 and 9 and lacks part of the DBD and the NLS (Panels A and B adapted from Marcel et al. 2011 [10]). (C) Overview of known biological functions of p53 protein isoforms. The green and red boxes indicate biological processes that are known to be either negatively or positively regulated by full-length p53, respectively. The different arrows indicate the type of regulation by the p53 isoforms.

In addition to full-length p53, the *TP53* gene physiologically expresses several protein isoforms, due to the use of alternative promoters, splicing sites, and translational initiation sites (Figure 1A) [10]–[12]. This constitutes an additional layer of p53 regulation concomitant to transcriptional, translational, and posttranslational regulatory mechanisms [10]–[12]. Two of the most characterized isoforms are Δ133p53α, which lacks the entire transactivation domain and part of the DNA-binding domain, and p53β, within which the oligomerization domain is replaced by 10 new amino acids (Figure 1B) [10], [11]. p53β has been shown to modulate p53 transcriptional activity in a promoter-dependent manner [13]. In contrast, Δ133p53α acts as a modulator of full-length p53 in response to stress, inhibiting p53-mediated apoptosis and G1 cell cycle arrest without inhibiting p53-mediated G2 cell cycle arrest. This suggests that Δ133p53α promotes p53-dependent cell survival in response to stress [14], [15]. Moreover, in normal human fibroblasts, Δ133p53α inhibits whereas p53β promotes p53-mediated replicative senescence [16]. An additional isoform has been described, Δp53, which lacks part of the DNA-binding domain and the nuclear localization signal (Figure 1B) [12], [17]. Δp53 was reported to be transcriptionally active toward specific p53 target genes and involved in the intra-S phase checkpoint in UV-damaged cells. However, the biological activity and relevance of this isoform remain controversial [13], [18]. Figure 1C shows a schematic overview of the reported biological functions of the p53 isoforms.

Although several studies report on the suppressive function of p53 isoforms and related deregulation of their expression in human cancers [18], investigations into the putative role of p53 isoforms and their regulation in pathogen infections have only recently begun. Three pioneer reports, including ours, have recently highlighted the role of p53 isoforms in epithelial cells infected by different pathogens: a gram-negative bacterium (*Helicobacter pylori*), a RNA, and a DNA virus (influenza and Simian virus 40, respectively) [19]–[21]. Despite major differences in terms of models and experimental strategies, these studies share some interesting preliminary conclusions regarding a new facet of p53 isoform biology.

**Figure 2:**
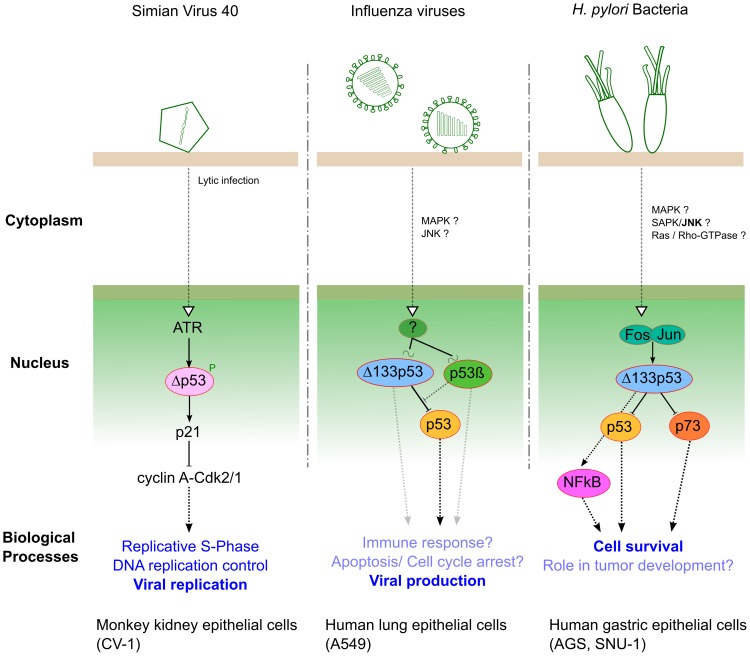
Three different pathogens represent three examples of interplay with p53 isoforms during infection.

Within SV40 lytic infection, p53 is targeted and inactivated by T-Ag, and Δp53 has been identified as a new player in SV40 replication by Rohaly and colleagues. They revealed that an ATR–DNA damage response pathway mediates the phosphorylation and stabilization of Δp53, enhancing its transcriptional activity in a promoter-dependent manner [19]. The activation of such an ATR–Δp53–p21 pathway results in down-regulation of cyclin A-Cdk2/1 (AK), maintaining the host cell in S-phase, which consequently favors viral amplification (Figure 2). Additionally, the same authors reported that the ATR–Δp53–p21 pathway also increases the subpopulation of host DNA polymerase α interacting with T-Ag, whose initiation is a prerequisite of origin-dependent viral replication [19].

In human lung epithelial cells, we have investigated the roles of both Δ133p53α and p53β isoforms in the context of influenza virus infection [20]. Our results have shown that infection differentially modulates the expression of Δ133p53α and p53β at both transcriptional and posttranscriptional levels. Reciprocally, we have revealed that the modulation of Δ133p53α and p53β isoforms play distinct roles in the viral cycle by acting as regulators of the p53-dependent antiviral activity (Figure 2). However, the upstream signaling cascade(s) and different downstream biological processes, both affected by cross-talk between full-length p53 and p53 isoforms, still require full characterization in the context of influenza infection.

Recently, *Helicobacter pylori* has been shown to interfere with p53 function via up-regulation of the Δ133p53 isoform both in vitro (gastric epithelial cells) and in vivo (Mongolian gerbil) [21]. Moreover, Wei and colleagues have identified the AP-1 transcription factor (cFos/cJun) as the upstream positive regulator of Δ133p53 transcriptional activity (Figure 2), leading to the suppression of both p53 and p73 functions and consecutively increasing cell survival. They also revealed that Δ133p53 is involved in the up-regulation of NF-κB in a p53-dependent manner, in the context of *H. pylori* infection (Figure 2). This study highlighted new and interesting ideas not only to decipher specific aspects of functional p53/NFkB antagonism but to better understand *H. pylori* pathogenesis and associated tumorigenesis.

In conclusion, these three studies, based on different pathogen models, have highlighted for the first time the functional role of different p53 isoforms in the context of infection. A preliminary model emerges in which the isoforms act as regulators of the p53-mediated cellular response against pathogens. As an illustration, both influenza viruses and *H. pylori* have an impact on Δ133p53 to interfere with full-length p53 activity via mechanisms remarkably similar to those previously described in the field of cancer [10]. However, the relative contribution of each p53 isoform in the hijacking of the p53 pathway by pathogens and/or the cellular antimicrobial response needs to be further explored. Based on these observations, we recommend that any future investigations focusing on the interplay between p53 and pathogens need to consider specific p53 isoforms, taking into account their different parameters such as relative ratios, chemical modifications, subcellular localizations, and tissue-specific expression. This new approach will certainly help to provide new insights into the multiple roles that p53 plays in pathogenesis, particularly by exploring the different biological processes involved, such as apoptosis, cell cycle, and immune responses.
